# Inhalable Dry Powders from Lyophilized Sildenafil-Loaded Liposomes with Resveratrol or Cholesterol as a Bilayer Component

**DOI:** 10.3390/ph19010129

**Published:** 2026-01-12

**Authors:** María José de Jesús Valle, Lucía Conejero Leo, David López Díaz, Amparo Sánchez Navarro

**Affiliations:** 1 Department of Pharmaceutical Sciences, Faculty of Pharmacy, University of Salamanca, 37007 Salamanca, Spain; 2 Institute of Biopharmaceutical Sciences, University of Salamanca (IBSAL), 37007 Salamanca, Spain; 3 Department of Physical Chemistry, Faculty of Pharmacy, University of Salamanca, 37007 Salamanca, Spain

**Keywords:** sildenafil, liposomes, resveratrol, dry powder, drug inhalation, pulmonary drug delivery

## Abstract

Pulmonary drug delivery represents a promising approach in the treatment of respiratory diseases, allowing for passive targeting and enhanced drug efficacy. **Background/Objectives:** The aim of the present study was to develop inhalable dry powders from lyophilized sildenafil citrate (SC)-loaded liposomes made from phosphatidylcholine and either cholesterol (CH) or resveratrol (RSV). **Methods:** Liposomes were prepared via a pH gradient method to increase drug entrapment efficiency and drug loading, and then the liposomes were lyophilized using different proportions of ethanol, mannitol, and lactose as excipients. The resulting dry cakes were converted into powders and evaluated for aerodynamic performance using a custom-designed air-blowing device. Notably, this is the first time that resveratrol has been used as a substitute for cholesterol in SC-loaded liposomes. **Results:** Our results demonstrate that RSV is a suitable liposome bilayer component and improves drug loading. Our findings prove that lyophilized cakes containing liposomes produce a dry powder that is suitable for aerosolization with potential application to pulmonary delivery of sildenafil citrate. The results suggest that RSV represents a potential alternative to traditional cholesterol-based liposomal formulations. **Conclusions:** This work presents a novel strategy for the pulmonary delivery of sildenafil, using biocompatible and FDA-approved mannitol and lactose for this administration route.

## 1. Introduction

A large proportion of the global population experiences various respiratory illnesses, with chronic obstructive pulmonary disease (COPD) being notably prevalent [1], ranking as the third leading cause of worldwide mortality. Asthma, tuberculosis, lung cancer, pulmonary hypertension (PH), and chronic bronchitis are also widespread. Due to the substantial global impact of these diseases, pulmonary drug delivery remains a significant public health challenge [2].

The inhalation route represents a non-invasive technique to deliver the drug directly to the lungs, thereby enhancing drug targeting and efficacy. This approach circumvents processes inherent in oral or intravenous administration, such as hepatic metabolism or active ingredient degradation along the gastrointestinal tract [3], and, most importantly, leads to fewer side effects related to systemic drug exposure. The pulmonary administration of drugs using nanoparticles through inhalers is increasingly gaining prominence as an alternative to traditional systems. Certain inherent characteristics of the lungs, including a vast absorption surface, exposure to high blood flow, a thin epithelial layer in the alveoli, and the slow turnover of the cellular surface, mean that inhalation represents a unique method of both systemic and local drug administration [4].

Medicine inhalation is typically achieved in the form of an aerosol, a suspension of fine droplets or solid particles in a gaseous medium, which can be produced by different devices, such as nebulizers, pressurized metered-dose inhalers (pMDIs), and dry powder inhalers (DPIs) [5]. Nebulizers are used in hospitals or for ambulatory chronic patients, as they are large and heavy devices; pMDIs and DPIs; however, are portable, and they contain the active ingredient suspended or dissolved in a volatile non-polar propellant and as a dry powder mixture, respectively. When comparing these two technologies, the latter exhibits greater stability, is more environmentally sustainable and does not require propellants. Another advantage is that DPIs can be used by patients who experience difficulties in coordinating device activation and simultaneous inhalation [6]. DPIs have proven to be more practical and efficient than pMDIs and nebulizers. Recent advancements have revealed other promising benefits, such as higher clinical efficacy in antibiotics, vaccines, and drugs showing poor oral bioavailability. Furthermore, DPIs allow for rapid action due to efficient local delivery, minimizing the dose required and the adverse effects caused by systemic drug exposure [1].

The development of systems to deliver drugs through the mucosa is an area of significant interest. Mucosa secretion is characterized by viscosity, elasticity, and stickiness, allowing tissue protection by trapping and eliminating foreign particles. These features hinder drug retention at mucosa body sites, but recent studies have proven the feasibility of designing nanoparticles that can overcome mucosal barriers [7] and have revealed the potential of liposomes in particular for this purpose. Liposomes are spherical lipid vesicles primarily composed of phospholipids and cholesterol. They form a lipid bilayer structure entrapping water, with an amphipathic domain [8]. Since their discovery, they have been extensively studied as drug carriers in the diagnosis or treatment of various pathologies [2]. These systems have the capacity to encapsulate drugs with varying solubility, both in their aqueous core and/or within the lipid bilayer [9]. Furthermore, due to their composition, these vesicles cross biological barriers, enhancing drug absorption, distribution, and targeting. In the particular case of the respiratory mucosa, the surfactant fluid in the upper respiratory pathways primarily consists of phospholipids, specific proteins, and other lipids [10]. The use of phospholipids as the principal components of liposomes confers numerous advantages in pulmonary delivery [11]. Phospholipids (marketed as substitutes for pulmonary surfactants) are biocompatible and biodegradable excipients that can enhance the migration of inhaled particles to peripheral lung regions [12,13,14]. Accordingly, liposomal formulations are proposed as ideal candidates for pulmonary drug administration, offering superior safety profiles, reduced macrophage clearance, and sustained drug release [15].

PH is a heterogeneous disorder that can lead to other pathologies, such as right ventricular failure and even death, in both adult and pediatric patients. It can have an idiopathic nature or be related to other pathologies, and its impact and incidence grow every day in the pediatric population, while treatment options remain limited [16]. Sildenafil citrate is a phosphodiesterase type 5 inhibitor (PDE-5) used to treat PH in adults and children, exerting vasodilator effects in the lungs [17]. PDE-5 is a modulating molecule in the nitric oxide/cyclic guanosine monophosphate (NO/cGMP) pathway. NO promotes pulmonary vasodilation through cGMP, which is broken down by PDE5, an enzyme found in the smooth muscle of the pulmonary arteries, which contributes to the development of PH [18]. Therefore, PDE5 inhibition can reduce PH by prohibiting the proliferation of smooth muscle cells and promoting apoptosis [19]. Recent research has confirmed its efficacy and safety in various patient groups, including those with PH secondary to connective tissue diseases and congenital heart defects [20]. Moreover, long-term studies suggest that sildenafil can improve functional capacity and quality of life in patients with PH [21]. Despite these benefits, it is crucial to monitor side effects and adjust therapy to reduce unwanted drug effects.

Inhalable formulations of sildenafil have been investigated regarding their potential in drug targeting in the treatment of PH. Polymeric nanoparticles, such as those made from PLGA (poly-lactic-co-glycolic acid), have been assayed, and the results show that they protect sildenafil from degradation and allow prolonged drug release. In addition, high encapsulation efficiency and stability after lyophilization and nebulization have been observed [22]. Paranjpe et al. [23] developed lipid formulations of sildenafil designed for DPI devices. These solid lipid nanoparticles were prepared using a novel microchannel homogenization method. Similarly, Makled et al. [24] produced solid lipid nanoparticles for the pulmonary delivery of sildenafil, demonstrating their potential in both nebulizers and DPI devices. Shahin et al. [25] developed large porous microparticles loaded with sildenafil using spray-drying techniques. These microparticles were specifically designed for DPI devices and showed controlled and sustained drug release in the lungs. More recently, highly porous iron-based metal–organic nanoparticles have been tested as drug carriers for the pulmonary delivery of sildenafil, and these particles were shown to be non-toxic in vitro and well tolerated in vivo [26]. However, SC liposome dry powders have not yet been investigated for applications in pulmonary delivery.

Due to the interest in liposomes as drug carriers for pulmonary delivery, the aim of the present study was to prepare sildenafil-loaded liposomes using either cholesterol (CH) or resveratrol (RSV) and egg phosphatidylcholine (EPC) as bilayer components and to produce inhalable dry powders from the lyophilized liposomes. This is the first time that sildenafil-loaded liposomes have been prepared using not cholesterol but resveratrol in the lipid bilayer. Another novelty of this work is found in the stabilization of liposomes via lyophilization and their transformation into inhalable dry powders using excipients specifically approved for drug inhalation by the FDA.

## 2. Results and Discussion

### 2.1. Liposomes

Higher entrapment efficiency and drug loading were observed for RSV liposomes (EE = 95.36 ± 3.87% and DL = 9.58 ± 0.43%) compared to CH liposomes (EE = 90.44 ± 4.62% and DL = 9.15 ± 0.46%), with the differences showing statistical significance (*p* < 0.05). These results showed improvement compared to previous data [27] obtained for sildenafil citrate-loaded liposomes using EPC and CH as bilayer components. Moreover, this is the first time that sildenafil citrate has been encapsulated in liposomes made from EPC and RSV instead of CH. It is preferable to avoid the use of CH in formulations, and the inclusion of RSV in its place provides the liposomes with additional advantages linked to the antioxidant and other beneficial properties of RSV [28]. Literature data support the suitability of liposomes entrapping RSV [29,30] but, so far, no data have been published supporting the viability of RSV/EPC liposomes as drug carriers; thus, this study is filling a key research gap.

**Figure 1:**
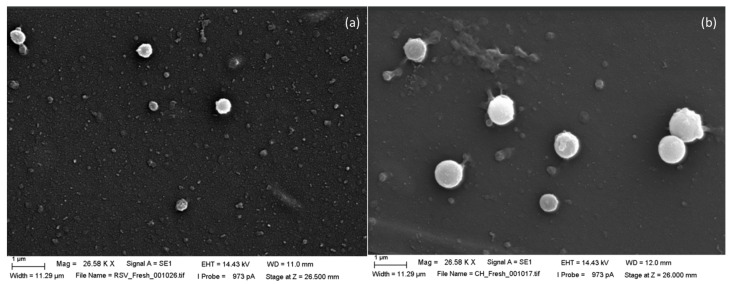
SEM images corresponding to fresh sildenafil-loaded liposomes prepared with RSV and EPC (**a**) or CH and EPC (**b**) as bilayer components.

Figure 1 shows SEM images of SC-loaded fresh liposomes prepared using RSV and EPC or CH and EPC as bilayer components.

**Figure 2:**
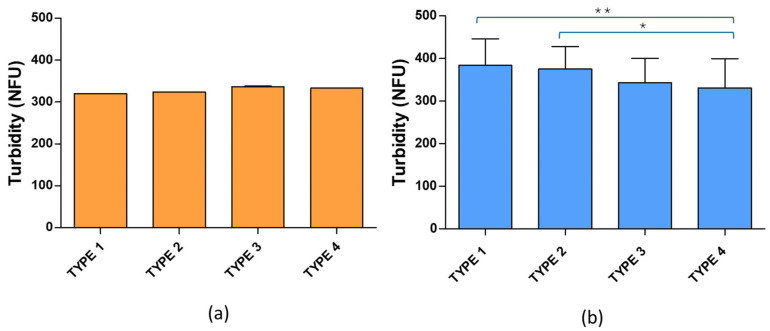
Turbidity mean values for resveratrol (**a**) and cholesterol (**b**) fresh liposome samples across four different compositions (Type 1: 2% ethanol, 1.5% lactose and 1% mannitol; Type 2: 2% ethanol, 3% lactose and 1.5% mannitol; Type 3: 1.5% lactose and 1% mannitol; Type 4: 3% lactose and 1.5% mannitol). ** *p* < 0.01; * *p* < 0.05.

Turbidity, that depends on the size and number of particles in suspension [31,32], as well as DLS data were considered in this study to compare the liposome samples. Figure 2 shows the turbidity results corresponding to fresh SC liposomes made from EPC and RSV or EPC and CH.

As shown in Figure 2, the turbidity mean values were homogeneous for fresh RSV liposome samples regardless of additives used for lyophilization. These samples displayed consistent turbidity across all four types, and statistical tests showed no significant differences among the four types (*p* > 0.05). Regarding CH liposomes, ethanol produced changes in turbidity, with statistically significant differences between samples with and without ethanol regardless of the amount of mannitol and lactose. Moreover, lower turbidity values were observed for RSV liposomes than for CH liposomes, pointing to the former being smaller than the latter. These results are in accordance with previous published data showing a larger hydrodynamic diameter for CH liposomes [27] compared to RSV liposomes [30]. These results are also in accordance with DLS data obtained in this study shown in Table 1 that includes mean values of Dh, PDI and zeta potential obtained for fresh and rehydrated liposomes. As expected, fresh RSV liposomes were smaller than CH liposomes with mean Dh values of 196.11 ± 5.98 nm and 289.80 ± 13.08 nm, respectively. Regarding zeta potential, negative values were observed in any case, with no statistically significant differences between RSV and CH liposomes (*p* = 0.1968), irrespectively of additives.

While it is well accepted that CH optimizes van der Waals packing and condenses the tail region of the lipid bilayer, there are controversial data for RSV. Published results dealing with the effect exerted by resveratrol on model membranes showed that RSV mainly locates in the hydrophobic core of the membrane [33], but other authors reported RSV interfacial location [34]. The differences between RSV or CH interactions with phospholipids may be responsible for the differences between RSV/EPC and CH/EPC liposomes in terms of entrapment efficiency and excipient effects on liposome characteristics.

### 2.2. Lyophilization

The conditions described in Material and Methods were applied for lyophilization and these are based on previous studies carried out with liposomes made from EPC and CH [35], with slight differences related to freezing and drying periods.

**Figure 3:**
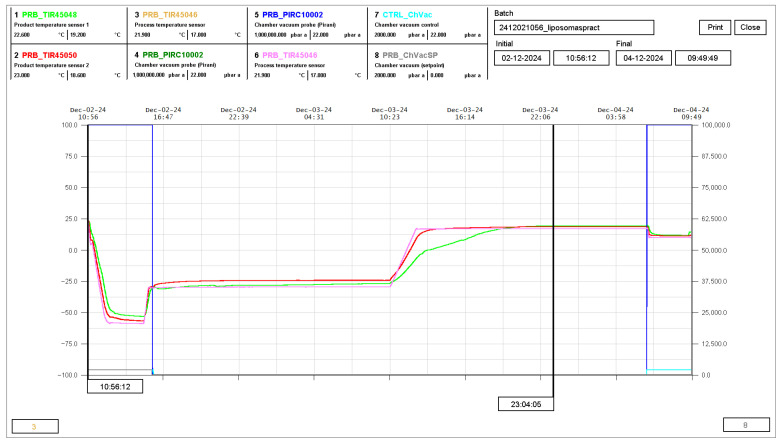
Display of registered curves corresponding to process (pink), CH liposome samples (green) and RSV liposome samples (red), together with chamber pressure (blue).

Figure 3 shows the registered curves for process and product temperature, as well as chamber pressure, over the course of the cycle. Superimposed temperature curves were registered for RSV and CH samples, which indicate the equivalent thermodynamic properties for both types of liposomes under freezing and drying conditions.

Smart and homogeneous cakes were obtained in all cases, regardless of RSV or CH liposomes, mannitol or lactose amount and the presence or absence of ethanol in samples. The remaining moisture was measured using Karl Fisher titration, with values in the range of 0.6–3.3%, irrespective of sample composition.

**Figure 4:**
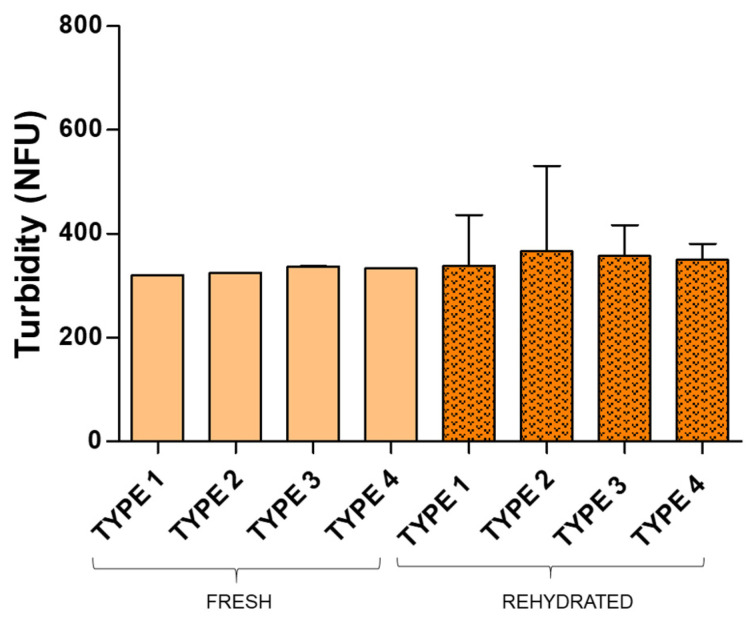
Turbidity median values for fresh and rehydrated samples containing resveratrol (Type 1: 2% ethanol, 1.5% lactose and 1% mannitol; Type 2: 2% ethanol, 3% lactose and 1.5% mannitol; Type 3: 1.5% lactose and 1% mannitol; Type 4: 3% lactose and 1.5% mannitol).

**Figure 5:**
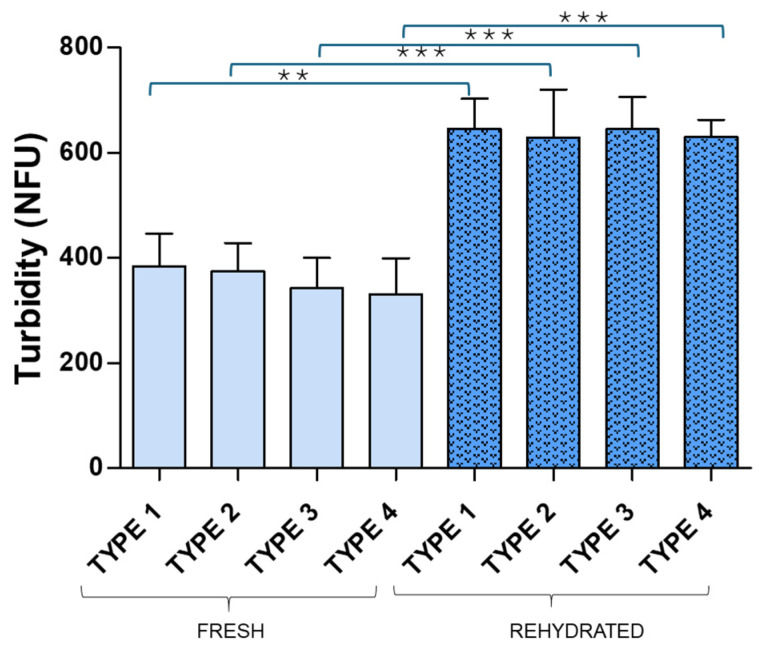
Turbidity median values for fresh and rehydrated samples containing cholesterol liposomes (Type 1: 2% ethanol, 1.5% lactose and 1% mannitol; Type 2: 2% ethanol, 3% lactose and 1.5% mannitol; Type 3: 1.5% lactose and 1% mannitol; Type 4: 3% lactose and 1.5% mannitol). *** *p* < 0.001; ** *p* < 0.01.

All samples were rehydrated, and the turbidity, Dh, PDI and zeta potential were measured. Figure 4 and Figure 5 illustrate the turbidity results obtained for rehydrated cakes compared to the corresponding fresh samples and Table 1 shows the DLS results for fresh and rehydrated samples.

In the case of RSV liposomes, statistically significant differences between fresh and rehydrated samples were not found, although higher dispersion for turbidity values was observed among rehydrated samples. For CH liposomes, however, turbidity median values increased for rehydrated samples compared to fresh ones, with statistically significant differences in all cases, these results pointing to potential vesicle aggregation. This effect has been widely reported in the literature for lyophilized liposomes [36,37], and the selection of cryo-lyo protectants to avoid liposome aggregation is a hot topic in current research [38,39]. Turbidity results obtained in this study suggest that vesicle changes produced by lyophilization were more evident for CH liposomes than observed for RSV liposomes, irrespectively of excipient used (30–40% turbidity increase was observed for rehydrated CH samples while less than 8% was observed for rehydrated RSV samples). DLS data, however, did not confirm this prediction but similar changes were observed for RSV and CH liposomes. A significant increase in Dh and PDI of rehydrated liposomes was observed for both RSV and CH liposomes in samples with ethanol. Samples without ethanol underwent less changes and those with the highest amount of mannitol and lactose showed the best results in terms of Dh, PDI and Zeta potential maintenance. For the latter, statistically significant differences between fresh and rehydrated liposomes were not found, irrespective of RSV or CH liposomes (*p* = 0.7488 and *p* = 0.8728, respectively) (Table 1). Despite turbidity being considered a surrogate marker of particle size, Dh and PDI values determined by DLS are more reliable parameters for characterization of liposome size distribution and these prove that 1.5% mannitol and 3% lactose successfully protected both RSV and CH liposomes during freezing-drying. These results are of particular relevance in the field of pulmonary drug delivery since mannitol and lactose are FDA approved excipients for drug inhalation. Moreover, the low amounts of excipients used here comply with the recommendations for pulmonary dry powder administration.

### 2.3. Dry Powders

**Figure 6:**
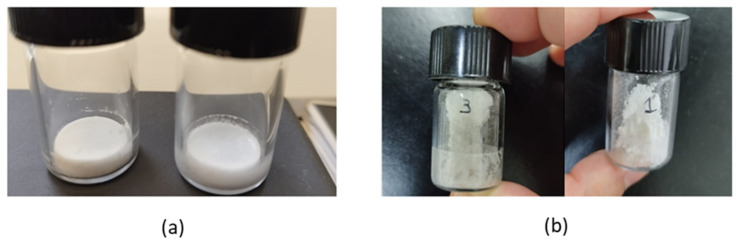
Lyophilized cakes (**a**) and corresponding dry powders (**b**) obtained via orbital agitation.

Powders with different aspects and characteristics were obtained from freeze-dried cakes with different compositions. Those obtained from undiluted samples were sticky, suggesting unfeasible aerosolizing under conditions simulating respiratory air turbulence. It was also observed that the addition of lactose carriers (230 Inhalac^®^ or 10 Inhalac^®^) before or after orbital agitation did not facilitate cake disintegration; rather, the opposite occurred. Accordingly, these samples were not evaluated in terms of aerodynamic performance. Only cakes obtained from diluted samples without added carriers produced a fine powder showing a cascade movement; therefore, only these powders were assayed in terms of aerodynamic performance. Figure 6 shows the aspects of cakes made from RSV and CH lyophilized liposomes (a) and their corresponding dry powders (b).

**Figure 7:**
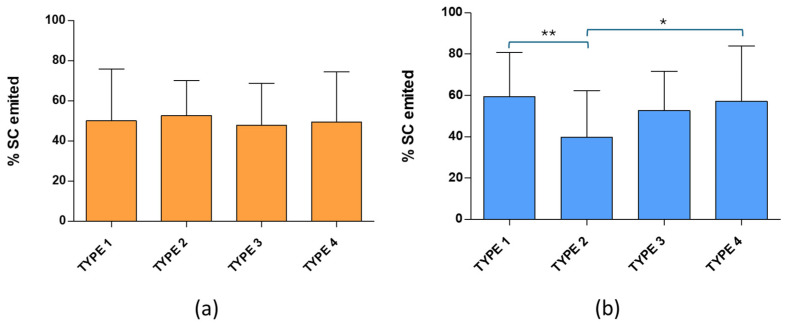
Percentage of sildenafil citrate (SC) emitted from dry powders containing resveratrol liposomes and cholesterol liposomes ((**a**) and (**b**), respectively). (Type 1: 2% ethanol, 1.5% lactose and 1% mannitol; Type 2: 2% ethanol, 3% lactose and 1.5% mannitol; Type 3: 1.5% lactose and 1% mannitol; Type 4: 3% lactose and 1.5% mannitol). ** *p* < 0.01; * *p* < 0.05.

The performance of powders was evaluated by comparing the drug amount measured in the samples containing the expelled powder with the drug amount in the original aerosolized cake. The percentages of SC emitted are shown in Figure 7. Median values were compared because the data did not show normal distribution. The expelled amounts were in the range of 42–53% and 33–60% for RSV and CH samples, respectively. More homogeneous and consistent results were observed for RSV liposomes, which did not show statistically significant differences among the four types of samples. For CH liposomes, however, statistically significant differences were found between Type 1 and Type 2, as well as between Type 2 and Type 4.

One of the main limitations of our study is the use of a device that does not provide internationally accepted metrics. Therefore, our results are just indicative of the potential of lyophilized powders for drug inhalation. The use of next-generation impactors (NGIs) is mandatory in the field of pharmaceutical aerosol characterization and this will be the next step for addressing and overcoming the limitation of our work in future studies.

According to the literature data, RSV seems like a molecule more versatile than CH in terms of location and interaction with bilayer lipids. This might justify the higher consistency of results observed for RSV liposomes along the study.

A previous study performed to evaluate the cytotoxicity of RSV liposomes confirmed cell viability in presence of RSV liposomes [30]; therefore, the present study was focused on the use of RSV instead of CH as a bilayer component of sildenafil loaded liposomes and also on the lyophilization of those liposomes to produce a dry powder suitable for inhalation. In vitro drug release as well as deposition studies are necessary to confirm the suitability of these lyophilized liposomes for pulmonary delivery of sildenafil citrate.

It is worth highlighting the variability of data obtained for the expelled fraction. Despite the high variability in the fraction of SC emitted, representing one of the limitations of our study, our results are in line with the literature data related to DPI formulations. Extensive research has been carried out to enhance the performance of dry powder inhalers by reducing variability and improving delivery efficiency [40]. Values for the total emitted dose in the range of 21.4–76.6% have been reported for terbutaline from DPI under different conditions, and similar or even lower values have been found for other drugs [41]. This is one of the obstacles still to be overcome in the field of pulmonary drug delivery, and numerous studies are currently focusing on this topic [42].

## 3. Materials and Methods

### 3.1. Materials

Egg yolk L-α-phosphatidylcholine and cholesterol were purchased from Sigma-Aldrich^®^ (Merk KGaA, Darmstadt, Germany). Laboratory HPLC-grade methanol was supplied by Thermo Fisher Scientific (Waltham, MA, USA). H_2_PO_4_, NaOH, and K_2_HPO_4_ were purchased from Panreac AppliChem (Darmstadt, Germany). Ultrapure water was obtained with a Wasserlab Automatic Plus System. Lactose monohydrate and mannitol were purchased from Guinama S.L.U. (Valencia, Spain). Sildenafil citrate, resveratrol, and triethanolamine were supplied by Acofarma (Madrid, Spain). Pure ethanol was purchased from LabKem, Labbox Labware (Migjorn, Barcelona, Spain). Lactose carriers (230 Inhalac^®^ or 10 Inhalac^®^ Lactose) were kindly provided by Meggle (Wasserburg, Germany).

### 3.2. Liposome Preparation and Characterization

Transmembrane pH gradient liposomes were prepared following a previously described method [35] with specific adaptations. In short, EPC with CH or RSV (0.7/0.3 EPC/CH or EPC/RSV molar ratio) were mixed with 1 mg/mL sildenafil citrate (SC) in buffer solution (pH = 4.7) to a total lipid concentration of 0.9% *w*/*w*. The resultant dispersion was transposed into a flask and subjected to ultrasonic agitation (Fisher Scientifics FB 15061, 50 Hz) (Fisher Scientific, Waltham, MA, USA) for 20 min at 40 ± 2 °C in the case of CH liposomes and at 37 ± 2 °C for RSV liposomes. Subsequently, the resulting suspension underwent eight rounds of filtration using syringe filters (Chromafil^®^ PET) (Macherey Nagel, Düren, Germany) with a pore size of 0.45 µm. Next, the mixtures were kept for 1 h at room temperature and then refrigerated for 1 h at 4 °C. Finally, NaOH was added to adjust the pH to 7.0, and samples were kept under agitation for 20 h at 4 °C to allow the unionized drug to cross the lipid bilayer and accumulate in the form of ionized molecules in the water core (pH = 4.7). Both type of SC-loaded liposomes (RSV/EPC and CH/EPC) were lyophilized and processed to obtain dry powders to be assayed for aerodynamic performance. Rigorous light protection protocols were observed for all samples containing RSV due to its inherent photosensitivity.

The turbidity of liposome suspensions was measured using an HACH 2100Q turbidimeter (Hach Company, Loveland, CO, USA) calibrated with standard samples in the range of 10–800 NTU (Nephelometric Turbidity Unit). Samples were properly diluted to ensure turbidity within the calibration range and inserted into the portable HACH 2100Q turbidimeter. To estimate the entrapment efficiency (EE) and drug loading (DL), the liposome suspensions were centrifuged for 90 min at 14,000 rpm and 4 °C. The supernatant was separated and diluted 1:10 with ethanol. SC was quantified in both the liposome suspension (previously diluted and vortexed) and the supernatant. The quantification of SC in samples was carried out via a high-pressure liquid chromatography (HPLC) assay. A Purospher^®^ STAR RP-18 end-capped C18 column (25 cm × 4.6 mm i.d., 3 μm particle size, 80 A pore size) was used. A 70% HPLC formic acid (0.1%) and 30% methanol mobile phase was applied at a 1.5 mL/min flow rate and a run time of 3 min. The column and sample temperature was 25 °C. The diode array detector was operated at 292 nm. The injection volume was set at 10 μL, and the samples were diluted and shaken with ethanol before HPLC injection. The calibration range was 5–300 μg/mL.

EE and DL were estimated according to the following equations:

(1) EE (%)=((Ct−Cs)/Ct)×100

where Ct is the total SC concentration (quantified in the liposome suspension) and Cs is the SC concentration quantified in the supernatant:

(2) DL (%) = (Csc/(Clip+Csc))×100

where Csc is the SC concentration in liposomes (1 mg/mL × EE) and Clip is the lipid concentration in the liposome suspension.

Hydrodynamic diameter (Dh), polydispersity index (PDI) and zeta potential of liposomes were determined by dynamic light scattering (DLS) using a Zetasizer Nano ZS (Malvern Instruments, Co., Malvern, UK). The analysis was performed at 25 °C and a scattering angle of 173° after the appropriate dilution of samples with MilliQ water to avoid multiple scattering.

Images of liposomes were obtained by scanning-electron microscopy (SEM) with thermionic emitters using ZEISS microscope Zeiss, (Oberkochen, Germany). Poly-l-lysine and osmium were used as fixing agents, ketone as the desiccant and gold for the metallic coating.

### 3.3. Lyophilization

**Figure 8:**
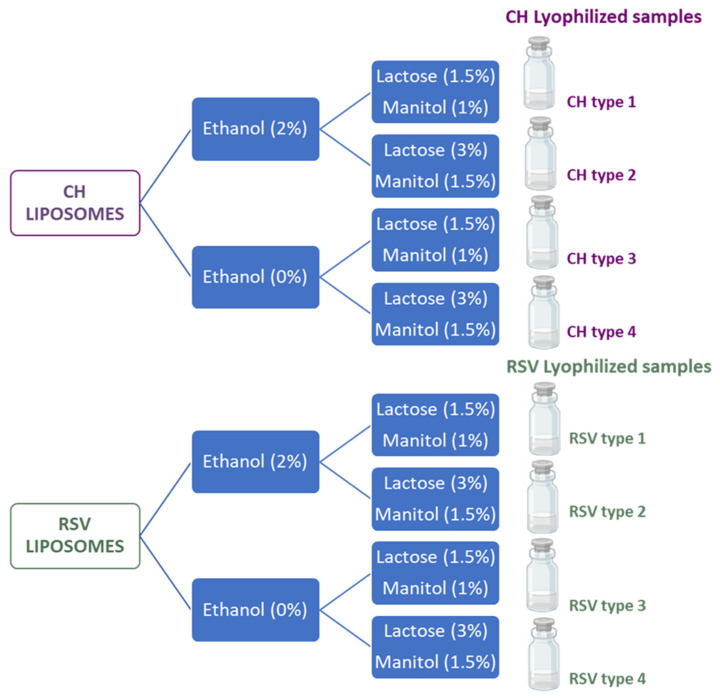
Schematic presentation of the experimental procedure applied to prepare liposome samples for lyophilization (CH, cholesterol; RSV, resveratrol).

Liposome suspensions were divided into 2 aliquots, and one was mixed with ethanol for a 2% (*w*/*w*) final concentration in order to increase the cakes’ porosity and ease of disintegration. As ethanol Pv (≈140 Pa at −40 °C) is higher than that of water Pv (≈10 Pa at −40 °C), the former solvent is quickly eliminated during early primary drying. Mannitol and lactose in different proportions were added to samples with and without ethanol. Figure 8 illustrates the experimental conditions applied to obtain lyophilized cakes for powder production.

Different volumes of the resulting mixtures (0.5–2 mL) were transferred to freeze-drying vials and lyophilized. After different lyophilization assays, 1 mL of liposome suspension diluted with Milli-Q water was selected as the best condition for sample lyophilization, and this condition was applied to the rest of the experiments.

A LYOBETA 4PS (Telstar) instrument (Telstar Cryodos, Telstar S.L., Terrassa, Spain), connected to LyoSuiteTM software (Lyosuitelab 3.0), was used for the lyophilization of liposomes. Freezing was performed at −60 °C and primary drying at −30 °C for 21 h, followed by secondary drying at 17 °C for 14 h, with a condenser temperature of −80 ± 4 °C and a chamber pressure of 25–50 µBar during the drying phases, to obtain the lyophilized cakes to be used for dry powder production. Temperature probes were placed in vials containing RSV and CH liposome samples.

Lyophilized samples were visually evaluated in order to check for puffing and/or sample collapse, as well as differences among sample types. The remaining moisture was determined using the Karl Fisher method, as follows: 0.1 g of dry cake was weighed using an analytical balance (Mettler Toledo, Columbus, OH, USA, XS105DU) and transferred to the titration cell. The water content was measured with the Metrohm 870 KF Titrino plus KF titrator (Metrohm, Herisaum, Switzerland).

Lyophilized cakes were rehydrated and samples were tested in terms of turbidity, Dh, PDI and Zeta potential as described above.

### 3.4. Dry Powders

The vials containing freeze-dried cakes underwent orbital agitation for different periods of time (1–5 h) at different speed rates (50–100 rpm), and the aspects of the resulting powders were observed. In order to evaluate the effects of lactose carriers (230 Inhalac^®^ and 10 Inhalac^®^), 30 or 60 mg was added to samples before or after orbital agitation, and the resulting mixtures were processed the same as those without the carriers.

Samples showing easy cake disintegration and producing homogeneous powders were selected for aerodynamic performance, with those showing agglomerates or sticky particles being excluded.

**Figure 9:**
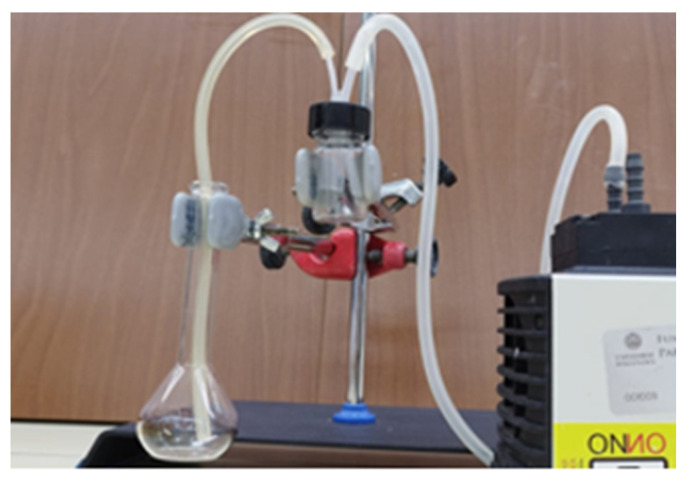
Device used for testing the aerodynamic performance of lyophilized dry powders. The device consists of an air pump, an in-out-tubing system, and a collecting flask for the expelled powder.

The aerodynamic performance of dry powders was assessed using a system inspired by the methodology outlined by Miyamoto et al. [43]. A home-produced device was used for this purpose. The system (Figure 9) includes a cap provided with in- and out-tubing, with the former connected to a pump generating a 1.67 mL/sec air flow, which entered the vial containing the dry powder, which was dragged through the outlet tube toward a flask containing water. After 3 s air blowing, the expelled amount of SC recovered in the flask was quantified using the HPLC assay described above.

The samples collected in the flask containing the expelled powder were also analyzed for turbidity, and the results were compared with those obtained for the corresponding lyophilized and rehydrated cakes.

### 3.5. Statistical Analysis

The results were recorded as mean or median values according to the distribution data shown in Figures S1–S5. The ANOVA test was used for normal distributed data, while the Kruskal–Wallis test with a multiple-comparisons Dunn test was performed for data without normal distribution. Statistical significance was considered for *p*-values ≤ 0.05.

## 4. Conclusions

In conclusion, the results of this study demonstrate the effectiveness of encapsulating sildenafil citrate in liposomes made from RSV and EPC, showing advantages compared to classic CH and EPC liposomes. The use of RSV instead of CH endows the liposomes with the beneficial properties of RSV, a finding that has not been previously reported. This work also shows the efficiency of a freeze-drying process using 3% lactose and 1.5% mannitol as protective agents in producing disintegrable cakes containing SC liposomes. Under the selected condition, lyophilized cakes were transformed into dry powders that produced expelled fractions in accordance with the literature data for PDI emitted doses. In summary, the results support the feasibility of utilizing lyophilized liposomes made from RSV and EPC as formulations for SC pulmonary administration. The entire procedure was carried out in the absence of organic solvents and using only FDA-approved additives for inhalable medicines as formulation components. Despite the positive results, further studies using in vitro models such as the Next-Generation Impactor to provide internationally accepted metrics are necessary. In addition, in vitro drug release as well as deposition assays must be carried out to confirm the viability and potential benefits of this proposal.

## Figures and Tables

**Table 1:** Dynamic Light Scattering results obtained for fresh and rehydrated liposomes lyophilized with different excipients (Type 1: 2% ethanol, 1.5% lactose and 1% mannitol; Type 2: 2% ethanol, 3% lactose and 1.5% mannitol; Type 3: 1.5% lactose and 1% mannitol; Type 4: 3% lactose and 1.5% mannitol).

		Dh (nm)	PDI	Zeta (mV)
RSV	Fresh	196.11 ± 10.98	0.23 ± 0.02	−39.3 ± 5.71
	Rehydrated Type 1	711.15 ± 297.20	0.74 ± 0.13	−40.70 ± 3.11
	Rehydrated Type 2	441.40 ± 24.82	0.51 ± 0.03	−47.00 ± 0.99
	Rehydrated Type 3	369.17 ± 16.05	0.55 ± 0.03	−38.6 ± 3.89
	Rehydrated Type 4	237.47 ± 25.39	0.35 ± 0.05	−41.90 ± 0.78
CH	Fresh	289.8 ± 13.08	0.37 ± 0.02	−43.7 ± 4.44
	Rehydrated Type 1	610.86 ± 30.90	0.29 ± 0.12	−44.90 ± 3.61
	Rehydrated Type 2	536.82 ± 53.39	0.35 ± 0.22	−44.80 ± 6.29
	Rehydrated Type 3	432.18 ± 91.00	0.50 ± 0.20	−45.20 ± 0.21
	Rehydrated Type 4	292.80 ± 5.0	0.36 ± 0.04	−42.60 ± 1.27

## Data Availability

The original contributions presented in this study are included in the article/Supplementary Material. Further inquiries can be directed to the corresponding authors.

## Supplementary Materials

The following supporting information can be downloaded at https://www.mdpi.com/article/10.3390/ph19010129/s1, Figure S1: Comparison of fresh RSV and CH liposomes; Figure S2: Comparison of fresh and rehydrated RSV liposomes; Figure S3: Comparison of fresh and rehydrated CH liposomes; Figure S4: Comparison of SC emission from RSV liposome dry powders; Figure S5: Comparison of SC emission from CH liposome dry powders.
