# Supplementary figures and images for: Inhalable Dry Powders from Lyophilized Sildenafil-Loaded Liposomes with Resveratrol or Cholesterol as a Bilayer Component
Source: Pharmaceuticals (Basel). 2026 Jan 12;19(1):129. doi: 10.3390/ph19010129 (PMC12844916; doi:10.3390/ph19010129)

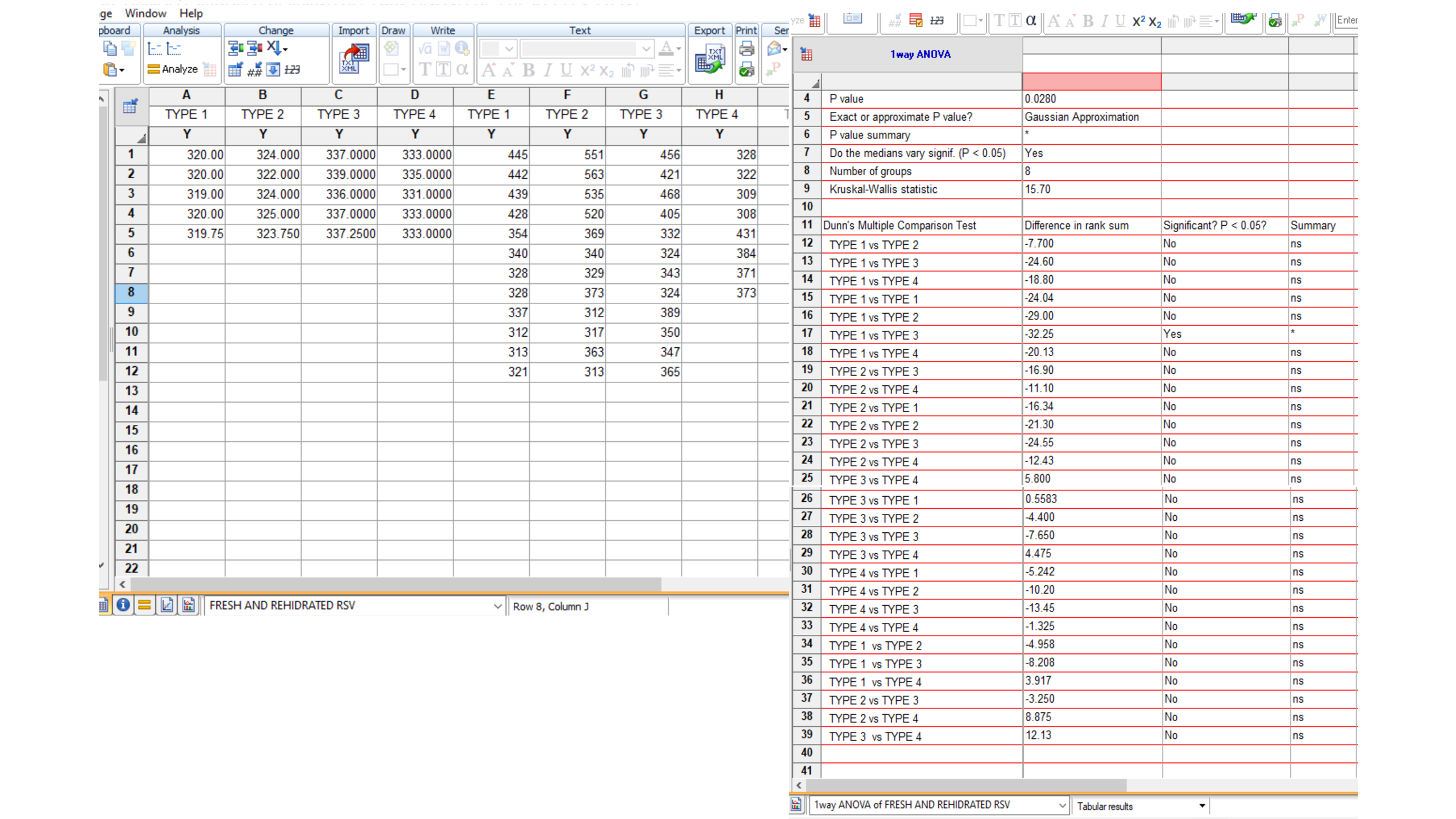

Supplement: Supplementary file 1 [file pharmaceuticals-19-00129-s001.zip › Figure S2.png]

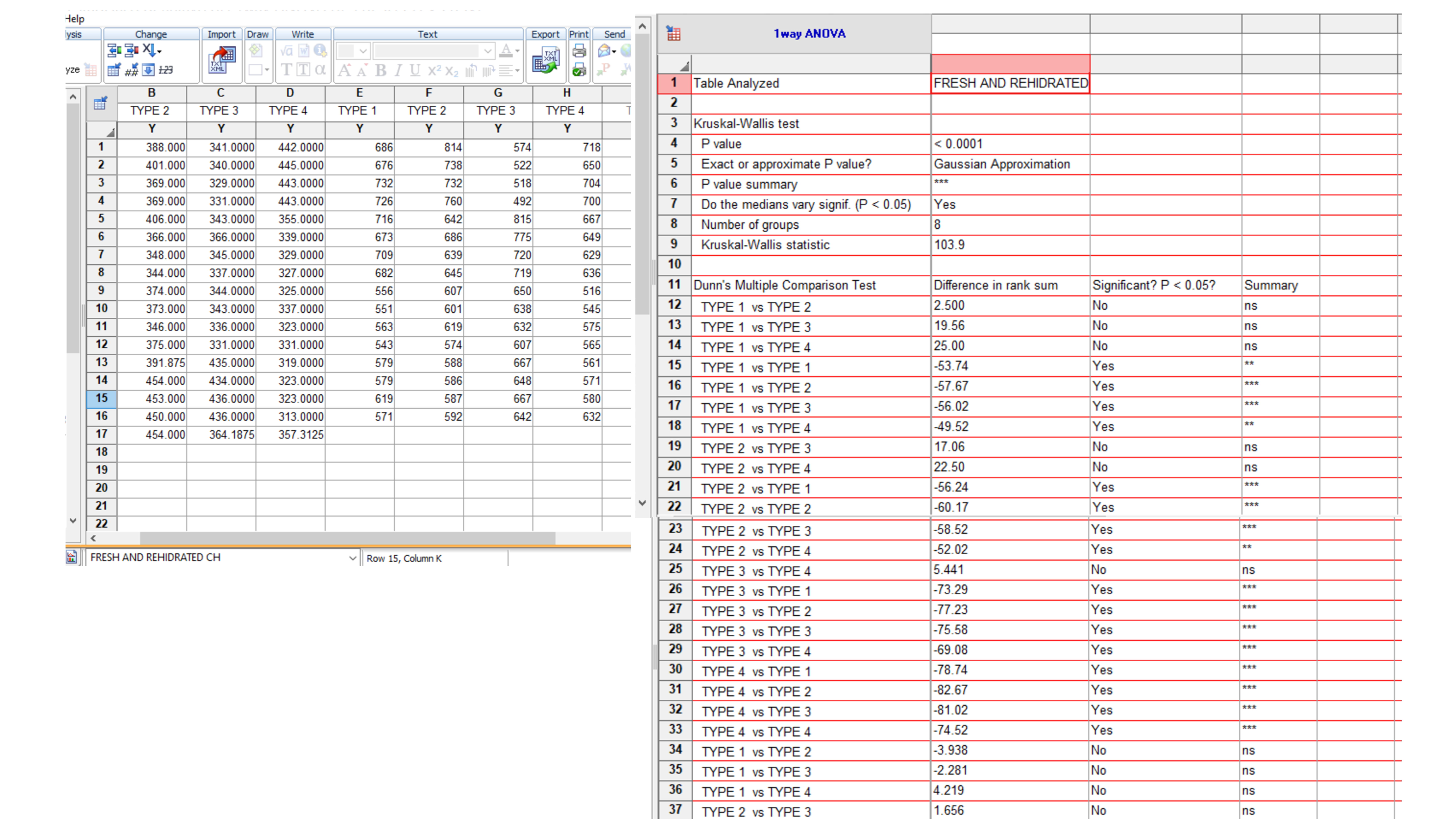

Supplement: Supplementary file 1 [file pharmaceuticals-19-00129-s001.zip › Figure S3.png]

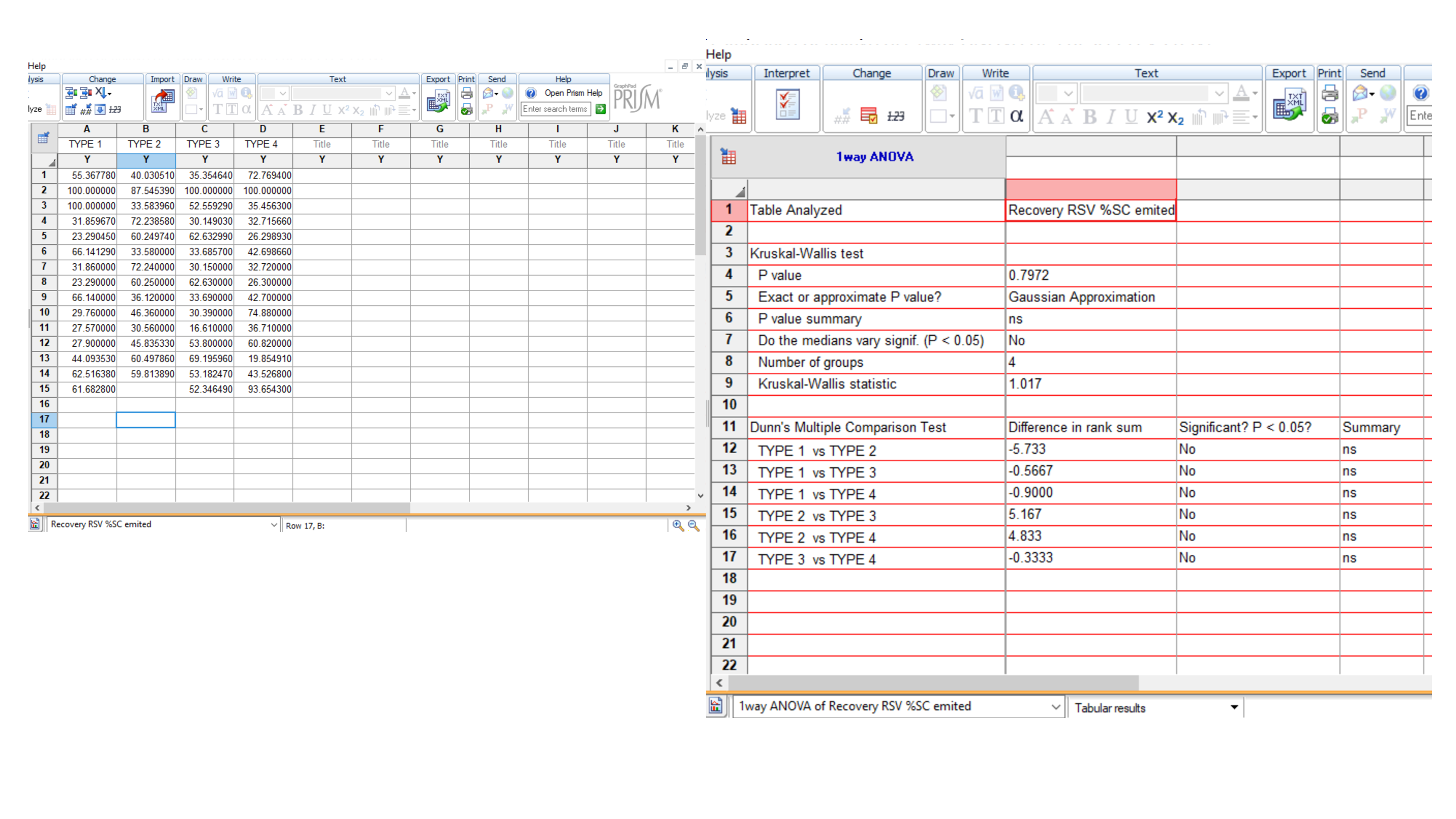

Supplement: Supplementary file 1 [file pharmaceuticals-19-00129-s001.zip › Figure S4.png]

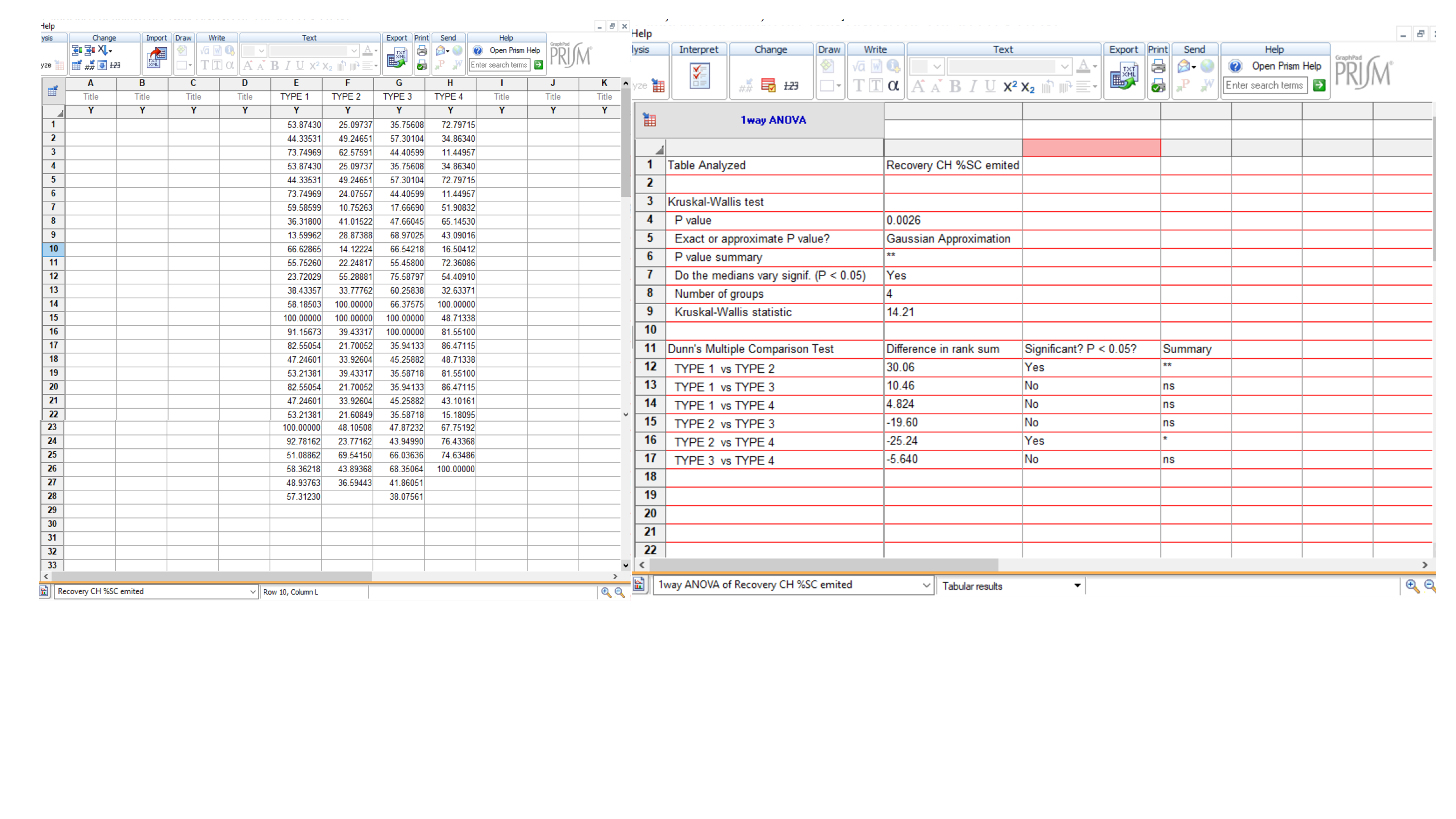

Supplement: Supplementary file 1 [file pharmaceuticals-19-00129-s001.zip › Figure S5.png]

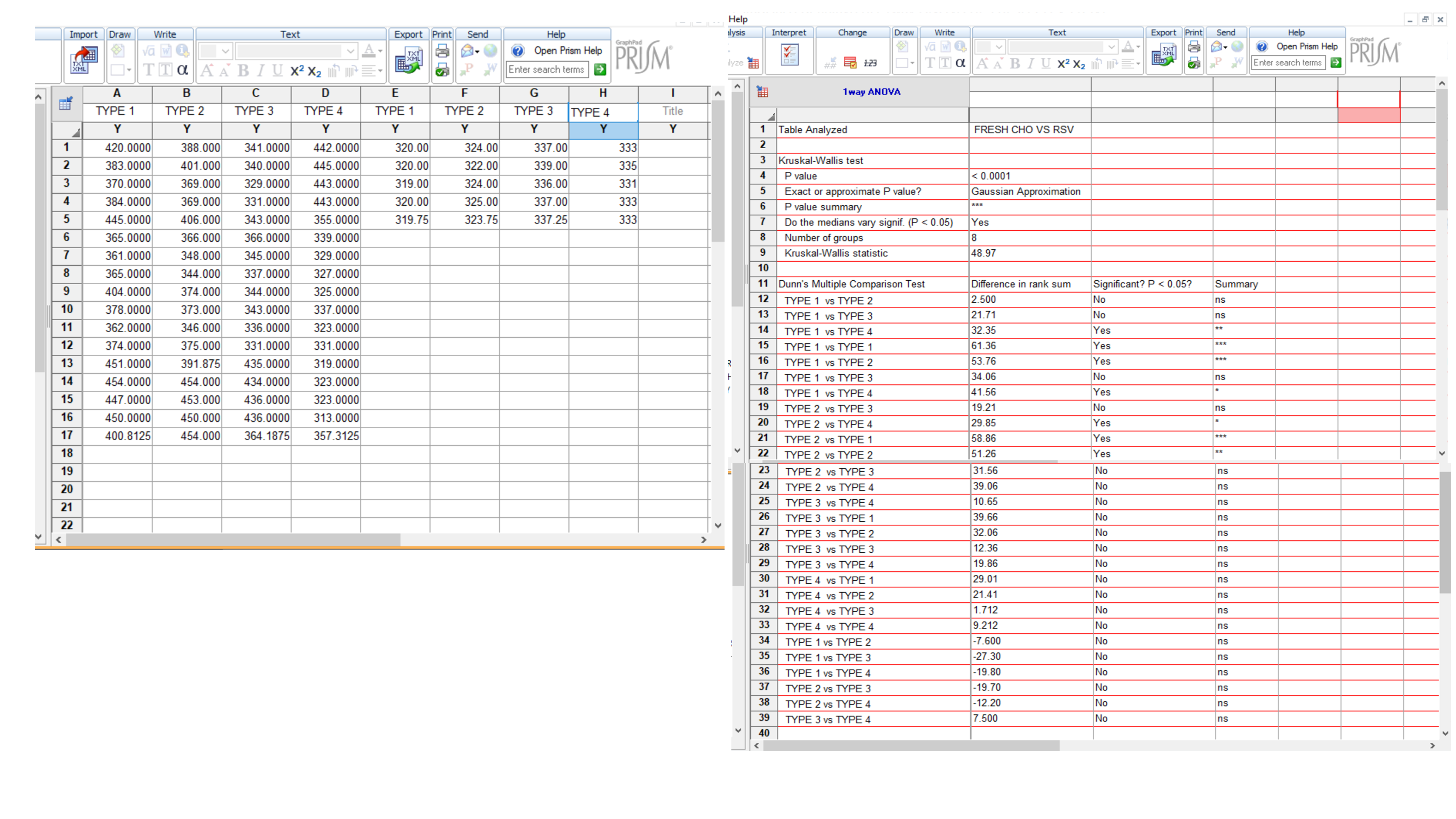

Supplement: Supplementary file 1 [file pharmaceuticals-19-00129-s001.zip › Figure S1.png]
